# Rapid Ocean Warming Drives Sexually Divergent Habitat Use in a Threatened Predatory Marine Ectotherm

**DOI:** 10.1111/gcb.70331

**Published:** 2025-07-16

**Authors:** Lucy R. Mead, Adam Piper, David Jiménez Alvarado, Eva Meyers, Joanna Barker, Hector Toledo‐Padilla, Edy Herraiz, Alberto F. Campoamor, Michael Sealey, Maria Belén Caro, Tomàs Bañeras, Charlotte Pike, Matthew Gollock, Felipe Ravina‐Olivares, David M. P. Jacoby

**Affiliations:** ^1^ Institute of Zoology Zoological Society of London London UK; ^2^ School of Biological and Behavioural Sciences Queen Mary University of London London UK; ^3^ Angel Shark Project Islas Canarias Spain; ^4^ IU‐ECOAQUA Universidad de Las Palmas de Gran Canaria Las Palmas Spain; ^5^ LIB, Museum Koenig Bonn, Leibniz Institute for the Analysis of Biodiversity Change Bonn Germany; ^6^ Conservation and Policy Zoological Society of London London UK; ^7^ La Graciosa Divers Islas Canarias Spain; ^8^ Lancaster Environment Centre Lancaster University Lancaster UK

**Keywords:** angelshark, behaviour, climate change, conservation, elasmobranch, intraspecific variation, marine predator, species distribution, thermal tolerance

## Abstract

Climate change poses one of the greatest threats to marine ecosystems worldwide, altering physical, chemical, and biological processes at unprecedented rates. Severe impacts on marine species and habitats have been extensively documented, with shifts in phenology, spatial distribution, and migratory behaviour increasingly pervasive. However, there is a lack of species‐specific data examining responses and adaptation to rapid warming and environmental extremes, especially for marine ectotherms. In this study, we investigate the broad‐scale environmental drivers of distribution in a Critically Endangered ectothermic marine predator, the angelshark *Squatina squatina*, and report on a behavioural anomaly observed in 2022. Between 2018 and 2023, 112 adult *S. squatina* were tracked using acoustic telemetry in La Graciosa Marine Reserve in the Canary Islands. Relationships between seasonal presence of *S. squatina* and remotely sensed environmental parameters were examined with Boosted Regression Tree and Generalised Additive Modelling. Major sex differences were found, with female sharks strongly influenced by environmental conditions and particularly sensitive to temperature, with a possible upper thermal threshold close to 22.5°C. Peak sea surface temperature in the study area increased from 22.99°C to 23.81°C, and the number of days above 22.5°C nearly tripled. Absence of females during the 2022 breeding season coincided with widespread thermal anomalies across the Northeast Atlantic Ocean, with unusually high temperatures persisting later into the year. We conclude that this potentially disrupted seasonal thermal cues for *S. squatina* movement, leading to sexually divergent habitat use. Given the warming projected for this region, thermal thresholds may increasingly be exceeded, and key areas may become inhospitable for female *S. squatina* , which is of huge concern for this already highly threatened species. These findings highlight the urgency of identifying species‐specific environmental tolerances and incorporating these into conservation so that management remains ecologically relevant in a rapidly warming ocean.

## Graphical abstract

How ectothermic, mobile marine predators are responding to climate change is uncertain due to lack of species‐specific data, which can mask the true impact of this threat. We investigate the impact of environmental conditions and thermal extremes on the behavior and distribution of angelsharks, a highly threatened elasmobranch of global conservation interest. Tracking angelsharks in the Canary Islands over 5 years revealed that females are more sensitive to thermal conditions than males. Persistent, widespread temperature anomalies in the 2022–2023 mating season led to sexually divergent habitat use, suggesting that this species is far more vulnerable to warming than previously thought.

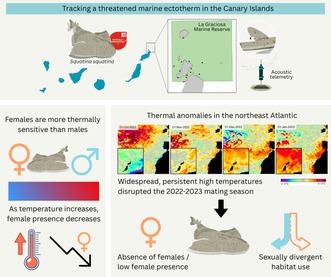

## ABSTRACTO

El cambio climático representa una de las mayores amenazas para los ecosistemas marinos a nivel global, al alterar los procesos físicos, químicos y biológicos a un ritmo sin precedentes. Se han documentado ampliamente impactos severos sobre especies y hábitats marinos, incluyendo cambios en la fenología, la distribución espacial y el comportamiento migratorio. Sin embargo, existe una carencia de datos específicos por especie que analicen sus respuestas y mecanismos de adaptación frente al calentamiento acelerado y a condiciones ambientales extremas, especialmente en ectotermos marinos. En este estudio, investigamos los impulsores ambientales a gran escala que determinan la distribución del angelote (Squatina squatina), un depredador marino ectotermo catalogado como En Peligro Crítico, y reportamos una anomalía comportamental observada en 2022. Entre 2018 y 2023, se monitorearon 112 ejemplares adultos de S. squatina mediante telemetría acústica en la Reserva Marina de La Graciosa, en las Islas Canarias. Las relaciones entre la presencia estacional de S. squatina y parámetros ambientales obtenidos por sensores remotos se analizaron mediante Árboles de Regresión Potenciados (BRT) y Modelos Aditivos Generalizados (GAM). Se encontraron diferencias marcadas entre sexos, con las hembras mostrando una fuerte influencia de las condiciones ambientales y una sensibilidad particular a la temperatura, con un posible umbral térmico superior en torno a los 22.5 °C. La temperatura superficial máxima del mar (SST) en el área de estudio aumentó de 22.99 °C a 23.81 °C, y el número de días por encima de 22.5 °C casi se triplicó. La ausencia de hembras durante la temporada reproductiva de 2022 coincidió con anomalías térmicas generalizadas en el Atlántico nororiental, donde las temperaturas inusualmente altas se mantuvieron avanzado el año. Concluimos que este fenómeno pudo haber alterado las señales térmicas estacionales que guían los movimientos de S. squatina, dando lugar a un uso del hábitat divergente entre sexos. Dado el calentamiento proyectado para esta región, es probable que se superen cada vez más los umbrales térmicos, volviendo inhabitables áreas clave para las hembras de S. squatina, lo que resulta alarmante para una especie ya altamente amenazada. Estos hallazgos destacan la necesidad urgente de identificar las tolerancias ambientales específicas de cada especie e integrarlas en las estrategias de conservación, asegurando así una gestión adaptativa y ecológicamente coherente frente a un océano sometido a un calentamiento acelerado.

## 1 Introduction

Understanding and projecting the impacts of anthropogenic climate change on the world's oceans is one of the most urgent and critical challenges of our time. Global mean sea surface temperatures (SSTs) have risen by 0.88°C since the beginning of the 20th century, and periods of anomalously high sea temperatures—or marine heatwaves (MHWs)—are increasing in frequency, severity, and duration (Hobday et al. 2016; Frölicher et al. 2018; IPCC 2022). In all oceans, these temperature increases are coupled with a plethora of complex oceanographic changes, including reduced marine oxygen availability, expansion of oxygen minimum zones (OMZs), increased salinity, altered wind stress patterns, and increased stratification of the upper ocean (Deutsch et al. 2015; Bindoff et al. 2019; IPCC 2022). The impacts of such changes on biological and ecological systems are complex and variable, especially in regions of particular oceanographic complexity.

The Northeast Atlantic Ocean is undergoing rapid warming, characterized by exceptionally high temperatures and record‐breaking MHWs; in June 2023, monthly SST anomalies up to 1.5°C warmer than average were recorded around the UK and Ireland, Iberian Peninsula, the Canary Islands, and northwest coast of Africa (https://climate.copernicus.eu/, https://www.mercator‐ocean.eu/). Wider oceanographic changes associated with warming in this region are influenced by features such as the Northeast Atlantic Eastern Boundary Upwelling System (EBUS) and associated Canary Current (Benazzouz et al. 2015; IPCC 2022), as well as the Atlantic Meridional Overturning Circulation (AMOC) current system, which contributes to climatology on a global scale (Olson et al. 2018; Mishonov et al. 2024). Driven by the Azores High pressure system and trade winds, seasonal upwelling and ocean stratification lead to high productivity and nutrient richness, characteristic of all EBUS zones (Pardo et al. 2011; Hernández‐Guerra et al. 2017; Bindoff et al. 2019; IPCC 2021). The Canary Current, a wind‐driven surface current flowing north to south between the Iberian Peninsula and Guinea‐Bissau coastline, is disrupted by the abrupt topography of the Canary Islands archipelago, causing turbulence and eddy formation downstream and interacting with upwelling (Barton et al. 2004; Hernández‐Guerra et al. 2017; Edo et al. 2019). While temperatures in other EBUS zones are generally predicted to cool, climate projections for the North Atlantic EBUS are highly uncertain (Pardo et al. 2011; Benazzouz et al. 2015; IPCC 2021). The region is also uniquely influenced by the Calima phenomenon, where Saharan desert dust is transported westwards across the North Atlantic and deposited in the ocean in huge volumes, enhancing productivity in the upwelling zone (Gallisai et al. 2014; Yu et al. 2019; van der Does et al. 2021; Rodríguez et al. 2023). While the frequency and intensity of Calima events is increasing with climate change due to aridity and desertification, the projected impact on SST is highly uncertain and spatially variable (Goudie and Middleton 2001; Foltz and McPhaden 2008; Gallisai et al. 2014; Francis et al. 2022). Collectively, the climatic characteristics of the Northeast Atlantic make it particularly challenging to understand and predict ecosystem responses to environmental change in this region, although significant impacts on fishes are documented in many studies (e.g., Perry et al. 2005; Vedor et al. 2021; Dahms and Killen 2023; Da Costa et al. 2024; Coulon et al. 2024).

Range shift is perhaps the most pervasive ecological impact, whereby organisms move to remain within optimal environmental conditions and/or to avoid suboptimal conditions, driven by direct physiological preferences and tolerances (Sunday et al. 2012; Hastings et al. 2020) or to track changes in prey distribution (Berg et al. 2010; Pinsky et al. 2020). Shifts usually occur latitudinally, with colonization and expansion at the leading (or poleward) range edge and extirpation and contraction at the trailing (or equatorward) range edge (Parmesan and Yohe 2003; Bates et al. 2014; Lenoir et al. 2020; Hastings et al. 2020; Pinsky et al. 2020). In marine environments, range can also shift by depth because deeper cooler waters provide thermal refugia, enabling behavioral thermoregulation through horizontal and vertical movement (Perry et al. 2005; Dahms and Killen 2023). However, the effectiveness of such species adaptation can vary greatly. For example, low species mobility and dispersal capacity limit potential for movement to escape adverse conditions or access refugia (Perry et al. 2005; Berg et al. 2010; Jones et al. 2013; Bates et al. 2014; Pinsky et al. 2020), and the occurrence of habitat stressors such as pollution and overfishing may limit the availability of suitable habitat to move into (Hare et al. 2016; Pinsky et al. 2020; Womersley et al. 2024). Phenological shifts are also expected, with disruption to the timing of key biological events such as breeding, spawning, and migration, which normally occur within a specific and narrow timeframe (Mills et al. 2013; McNamara et al. 2011; IPCC 2022). In many species, seasonal migration and space use are inherently linked to variability in environmental conditions and are usually cued or triggered by a change in the local environment (Brodersen et al. 2012; Schlaff et al. 2014; Winkler et al. 2014). As climate change impacts accelerate and intensify, formerly reliable and regular environmental cues may be disrupted, leading to mistiming of migration and mismatch between migratory behavior and habitat suitability (McNamara et al. 2011; Winkler et al. 2014).

At the individual level, an organism can tolerate some change in its immediate environment through behavioral plasticity, such as thermoregulation and short‐term avoidance of physiologically stressful conditions (Chin et al. 2010; Pinsky et al. 2020). This mechanism is especially critical for ectothermic species, for which thermal and chemical changes have a direct impact on physiology and behavior (Berg et al. 2010; Pinsky et al. 2020; Osgood et al. 2021; Godefroid et al. 2023). However, this response becomes inadequate if exceeded by the pace and magnitude of environmental change, as can be the case in extreme climatic events such as MHWs (Bates et al. 2014; Godefroid et al. 2023). In turn, short‐term extremes exacerbate the impact of decadal‐scale changes (Hobday et al. 2016; Oliver et al. 2018; Cheung and Frölicher 2020), especially in marine ecosystems where the geographic ranges of ectotherms tend to closely match limits of thermal tolerance, leading to lower thermal safety margins and higher thermal sensitivity than in terrestrial ectotherms (Berg et al. 2010; Sunday et al. 2012; Pinsky et al. 2019; Dahms and Killen 2023). At all scales, responses and vulnerability to climate change are therefore ultimately dependent on species‐specific environmental constraints and tolerances (Pinsky et al. 2020). However, such species‐specific information is often lacking for marine ectotherms, including many of the chondrichthyan fishes (sharks, skates, rays and chimaera) (Chin et al. 2010; Jones et al. 2013; Osgood et al. 2021), which themselves play important roles in marine ecosystem structure and function as top‐order predators (Baum and Worm 2009; Ferretti et al. 2010; Roff et al. 2016). Many chondrichthyans are severely threatened by overfishing and habitat loss, and climate change is expected to exacerbate existing threats, as has already been observed in some species (Dulvy et al. 2021; Williamson et al. 2024; Womersley et al. 2024). For example, warming‐driven shifts in the distribution and migration phenology of tiger sharks, *Galeocerdo cuvier*, in the Northwest Atlantic were shown to reduce overlap with spatial protections and increase exposure to fisheries (Hammerschlag et al. 2022). Given that more than a third of chondrichthyans are already threatened with extinction, addressing environmental data gaps for this group is a global marine conservation priority (Dulvy et al. 2021).

The angelshark, *Squatina squatina* , is a benthic, dorsoventrally flattened, large predatory elasmobranch (Meyers et al. 2017; Noviello et al. 2021). As an ambush predator, *S. squatina* spends a large proportion of its life buried in the sediment to rest and hunt. It is of significant conservation concern, being listed as Critically Endangered on the IUCN Red List of Threatened Species, due to drastic range decline driven by overfishing and coastal habitat degradation (Morey et al. 2019). Formerly widespread throughout shelf waters of the Northeast Atlantic and Mediterranean Sea, *S. squatina* is now thought to have isolated and fragmented populations in the Mediterranean, North Sea, and around the Canary Islands (Gordon et al. 2017; Lawson et al. 2020; Meyers et al. 2017). The Canary Islands are an especially important region for this species; both juveniles and adults are regularly sighted and recorded across the archipelago, such that *S. squatina* has become a flagship species in the local dive industry (Modino 2011; Meyers et al. 2017; Jiménez‐Alvarado et al. 2020). Research has recently shown that strong genetic differentiation occurs across the archipelago, with several distinct and geographically isolated *S. squatina* populations, potentially increasing vulnerability to local and regional extirpation (Meyers et al. 2024). Crucially, these Canary Island populations are at the southernmost extent—or trailing range edge—of *S. squatina* distribution, and therefore close to their upper thermal limit, with possibly lower tolerance for environmental change and greater potential for extirpation (Deutsch et al. 2015; Pinsky et al. 2019).

Studies have linked *S. squatina* presence to temperature‐related variables and productivity (Jones et al. 2013; Meyers et al. 2017; Noviello et al. 2021; Barker et al. 2022; Giovos et al. 2022), and in other angel shark species, associations with temperature, productivity, and salinity have been identified (Colonello et al. 2007; Vögler et al. 2008; Bunholi et al. 2022). Strong seasonality in *S. squatina* movement, distribution, and habitat use has been observed and generally linked to the breeding cycle, and in some cases, to environmental factors (Bom et al. 2020; Ellis et al. 2021; Barker et al. 2022). For example, it is hypothesized that the Canary Islands populations undertake seasonal inshore‐offshore migration, with breeding occurring in nearshore coastal waters in late autumn to early winter, and warmer months spent in cooler offshore waters (Meyers et al. 2017; Tuya et al. 2020; Noviello et al. 2021). Seasonal and spatial sexual segregation and sex‐specific habitat use have also been observed, with males more likely to utilize deeper offshore habitat and females occupying shallower nearshore habitat for a greater proportion of the year (Mead et al. 2023). The extent to which environmental conditions drive the seasonal distribution, breeding behavior, and inshore‐offshore movement observed in *S. squatina* , as well as how males and females might respond differently to these selective pressures, is unclear.

In this study, acoustic telemetry and satellite remotely sensed environmental data were used to investigate the role of environmental conditions on seasonal presence and movement of adult *S. squatina* in La Graciosa Marine Reserve (LGMR) in the Canary Islands. Using this model species occupying an equatorward range edge and in a region experiencing acute climatic changes, we explore and discuss marine ectotherm responses to rapid environmental change and the impact on sexually divergent reproductive strategies. The primary research objectives were:

- To determine the relative influence of different environmental parameters on the seasonal presence of *S. squatina* ;
- To identify sex differences in environmental–behavioural coupling;
- To determine temperature cues associated with seasonal movement and shifts in the distribution of *S. squatina* ;
- To explore interannual patterns of *S. squatina* presence and whether this relates to environmental trends, specifically in relation to widespread temperature anomalies in 2022.

## 2 Materials and Methods

### 2.1 Study Site

**FIGURE 1:**
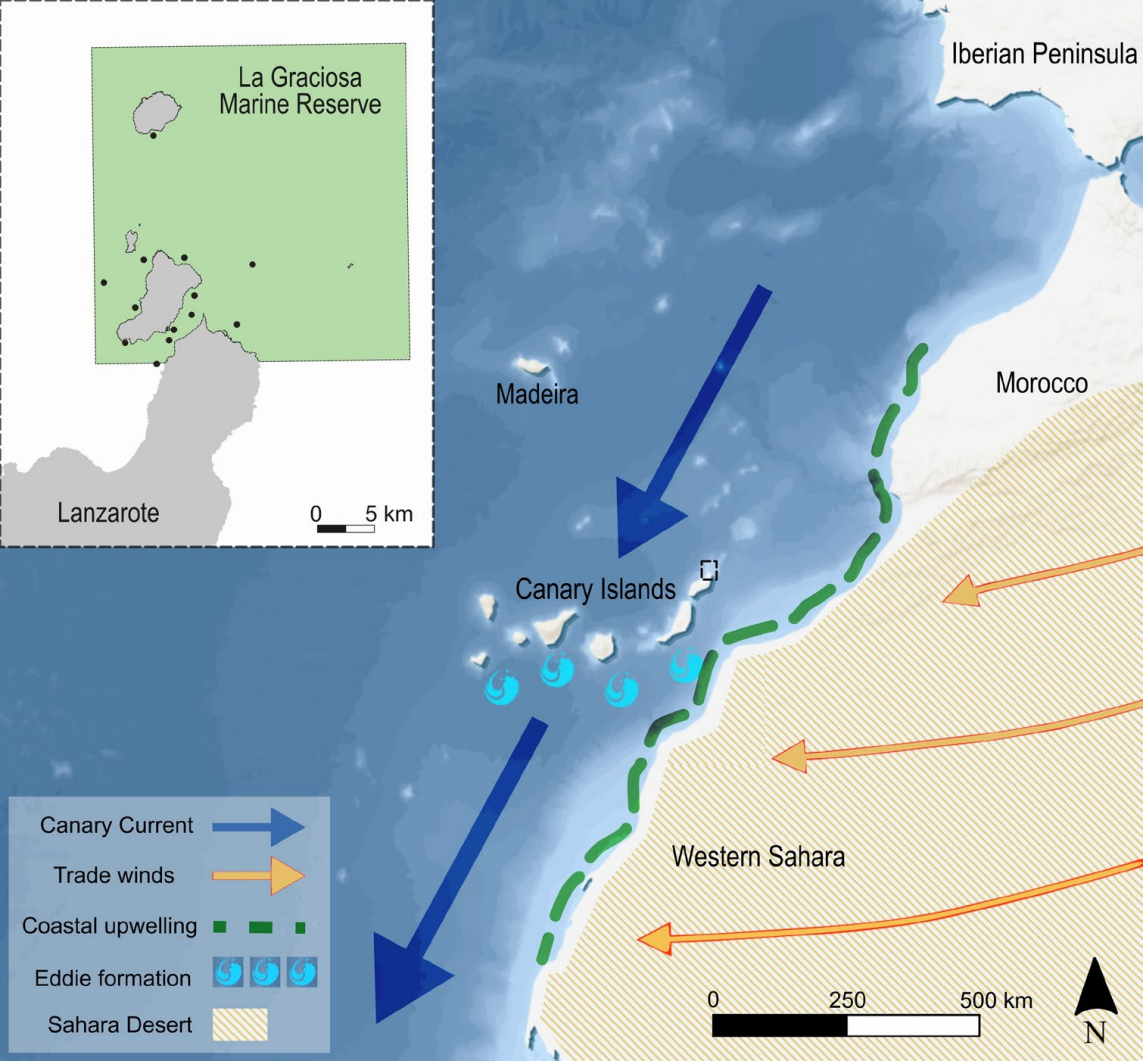
The Canary Islands and surrounding Northeast Atlantic region, indicating key geophysical and oceanographic features which influence climate and marine ecosystems, and the acoustic telemetry array and La Graciosa Marine Reserve, with black points indicating receiver locations and the green box indicting the marine reserve area (insert). Map lines delineate study areas and do not necessarily depict accepted national boundaries.

The Canary Islands are a Spanish archipelago in the Northeast Atlantic, consisting of eight main islands and five islets, distributed over a distance of almost 500 km from east to west (Figure 1). La Graciosa Marine Reserve (LGMR) encompasses one island and a series of islets situated to the furthest northeast of the Canary Islands and has an oceanic desert climate, characterized by low rainfall and northerly prevailing winds (Cordero et al. 2016). Covering an area of 70,764 ha, LGMR is Spain's largest marine reserve, and recreational and professional activities (e.g., fishing and SCUBA diving) are strictly controlled and regulated by the Spanish Government and Canary Islands Government (see Mead et al. 2023). *Squatina squatina* is not specifically included within a marine reserve management plan, although the species is protected throughout the Canary Islands under the Spanish Catalogue of Threatened Species (https://www.boe.es/eli/es/o/2019/04/08/tec596). The area is considered a hotspot for adult *S. squatina* , especially during the mating season (November to January) (Meyers et al. 2017; Mead et al. 2023).

### 2.2 Acoustic Telemetry

Continuous quantitative data on adult *S. squatina* presence in the study area were collected using an array of 14 acoustic receivers (Innovasea models VR2W, VR2Tx, and VR2AR). Receivers were deployed across the site between July 2018 and April 2023, in waters ranging from 10 to 130 m depth. Receiver range testing was carried out at each receiver using a hydrophone, test tag, or sentinel tags to determine the approximate distance at which acoustic transmissions were still detected. Acoustic tagging of *S. squatina* was carried out in November of each year from 2018 to 2022, and in July 2018 at the outset of the study. A bespoke tag applicator and novel underwater animal capture methodology allowed sharks to be tagged externally and in situ using SCUBA equipment, reducing stress and disturbance to the animal associated with capture and transfer to the surface. Individuals were opportunistically selected for tagging, and sex was determined through visual inspection of clasper presence or absence. Between 2019 and 2021, sharks were tagged with coded Innovasea V9 acoustic transmitters (*n* = 84), and from 2021 onwards with Innovasea V13 acoustic transmitters (*n* = 28). Both tag types were set to high power output and a 90–150 s delay. Based on manufacturer estimated battery life, V9 tags transmit for 346 days and V13 tags for 522 days. A total of 112 adult *S. squatina* (38 males and 74 females) were tagged during six tagging campaigns between July 2018 and November 2022 (Table 1). Further details on the tagging procedure and acoustic array design are available in Mead et al. (2023).

**TABLE 1:** Summary of acoustic tag deployment on male and female *Squatina squatina* over six tagging campaigns between July 2018 and November 2022.

Tagging campaign	Transmitter type	Total tags	Males tagged	Females tagged
Jul‐18	V9	9	2	7
Nov‐18	V9	13	5	8
Nov‐19	V9	32	10	22
Nov‐20	V9	30	6	24
Nov‐21	V13	20	9	11
Nov‐22	V13	8	6	2

### 2.3 Environmental Data

For environmental modeling, a range of remotely sensed and modeled variables were selected for investigation based on (i) known environmental associations in Squatiniformes and other elasmobranchs, (ii) specific local physical characteristics, and (iii) regional factors of interest in relation to climate change. Selected variables included sea surface temperature (SST), SST anomaly (SSTA), chlorophyll‐a concentration (as a proxy for productivity), salinity, dissolved oxygen (DO) concentration, sea surface wind speed, atmospheric dust aerosol, and atmospheric particulate matter (PM10); the latter two variables are proxies for desert dust and are used to forecast Calima events in the Copernicus Atmosphere Monitoring Service (CAMS) Aerosol Alert service (https://aerosol‐alerts.atmosphere.copernicus.eu/) (Foltz and McPhaden 2008; Querol et al. 2008).

All selected data had daily temporal resolution and Level 4 processing or equivalent (i.e., gap free, science quality). SST and SSTA data were sourced from NASA JPL's “GHRSST (Group for High Resolution Sea Surface Temperature) MUR Global Foundation Sea Surface Temperature Analysis” product (https://podaac.jpl.nasa.gov/dataset/hMUR‐JPL‐L4‐GLOB‐v4.1), and downloaded and extracted using R packages “rerddap” and “rerddapXtracto” (JPL MUR MEaSUREs Project 2015; Chin et al. 2010). Chlorophyll‐a concentration and wind speed data were sourced from the NOAA CoastWatch “VIIRS Ocean Color chlorophyll DINEOF gap‐filled analysis” (https://coastwatch.noaa.gov/cwn/products/noaa‐msl12‐multi‐sensor‐dineof‐global‐9km‐gap‐filled‐products‐chlorophyll‐diffuse.html) and “NOAA NCEI Blended Seawinds” (https://coastwatch.noaa.gov/cwn/products/noaa‐ncei‐blended‐seawinds‐nbs‐v2.html) products, respectively, and downloaded using ERDDAP Griddap Data Access Forms (Saha and Zhang 2022; Liu and Wang 2023). Salinity and dissolved oxygen concentration data were sourced from the Copernicus Marine Service (CMS) “Atlantic‐Iberian Biscay Irish Ocean Physics Analysis and Forecast” (https://data.marine.copernicus.eu/product/IBI_ANALYSISFORECAST_PHY_005_001/description) and “Atlantic‐Iberian Biscay Irish‐ Ocean Biogeochemical Analysis and Forecast” (https://data.marine.copernicus.eu/product/IBI_ANALYSISFORECAST_BGC_005_004/description) products, respectively (Sotillo et al. 2015; Gutknecht et al. 2019). Data for Calima dust—particulate matter *d* < 10 μm (PM10) and dust Aerosol Optical Depth at 550 nm—were sourced and downloaded from the CAMS global atmospheric composition forecast products (https://ads.atmosphere.copernicus.eu/cdsapp#!/dataset/cams‐global‐atmospheric‐composition‐forecasts?tab=overview) (Copernicus Atmosphere Monitoring Service 2021). Salinity, dissolved oxygen and Calima datasets were downloaded in raster format and the required data were extracted for the study site using the “Extract Raster Values” tool in QGIS S1.

### 2.4 Data Analysis

#### 2.4.1 Acoustic Detection Data

All data processing and analysis were carried out in R Studio (Version 4.4.1) and QGIS (Version 3.16.1). Acoustic data were filtered to remove erroneous detections and those associated with unrecognized tags, VR2Tx, or VR2AR internal transmitters, and range test tags (Mead et al. 2023). Detections within 48 h of tagging were removed to ensure that any unusual post‐tagging behavior did not influence the results. To explore the influence of wider Northeast Atlantic Ocean processes on *S. squatina* presence in the area as a whole, detection data were aggregated across all receiver locations. Total detection count data was reduced to “days detected” values for each individual, reflecting the number of days each shark was present in the study area (as per Mead et al. 2023). To investigate both seasonal and interannual variability in *S. squatina* presence, temporal patterns of acoustic data—as detection frequency or days detected—were plotted as a continuous time series for each individual and aggregated by month of the year. For environmental modeling and temperature cue analysis, detections were summarized as either binary presence/absence per day or number of individuals present per day.

#### 2.4.2 Environmental Modelling: BRTs and GAMs

To address study aims (i) and (ii), associations between *S. squatina* presence/absence in the study site and environmental drivers were investigated using Boosted Regression Tree (BRT) modelling. This technique allows for non‐normally distributed data and multiple complex interactions between variables. Due to the availability of environmental data, models were carried out for the period from April 2021 to April 2023, and as such only a subset of the acoustic detection dataset was included in this part of the analysis. Male and female presence was modelled separately in order to capture possible sex differences in environmental preference and association. The response variable in each model was daily binary presence/absence of males or females in the whole study site (where 1 indicates detection of one or more tagged sharks on any receiver on each day and 0 indicates no detected sharks). The explanatory variables included in the initial models were daily averages of SST, SSTA, chlorophyll‐a concentration, salinity, dissolved oxygen concentration, and sea surface wind speed, as well as day of year (doy) to account for seasonal variation. All models were fitted using the gbm.auto package (Dedman et al. 2017) in R, which automated the BRT process and generated marginal effects plots for each variable, as well as the relative influence of each variable on *S. squatina* presence/absence.

Optimal model parameters were found by testing different combinations of tree complexity (tc), learning rate (lr) and bagging fraction (bf) values for each sex. We tested tree complexity values of 2, 5, and 8 (to allow for binary interaction as a minimum and interactions between all eight variables as a maximum), various learning rate values between 0.0005 and 0.1 (0.0005, 0.001, 0.005, 0.01, 0.05, and 0.1), and bagging fraction values of 0.5, 0.6, and 0.7. The performance of models with 1000 trees or more was then evaluated based on mean deviance, relative deviance explained, and Area Under Curve (AUC) scores, and the potential for overfitting was assessed based on the difference between training and cross‐validation (CV) AUC scores (as per Dedman et al. 2017; Bangley et al. 2020, 2022). Based on the relatively low deviance explained values of this first round of models, we decided to investigate the possible influence of Calima by running a subset of BRT models with atmospheric dust aerosol concentration and PM10 as additional explanatory variables. For Calima models, parameter combinations were tested for learning rate values of 0.01, 0.005, and 0.001, and bagging fraction values of 0.5, 0.6, and 0.7. Models were assessed again to see if adding Calima variables improved performance. To control for potential collinearity between environmental variables, pairwise Pearson's correlation tests were carried out for all variable pairs S2. Where correlation was both significant (*p* < 0.05) and strong (*r* > 0.7), one correlated variable was dropped. If this did not improve model performance, all variables were retained in the final model selection.

Post hoc Generalised Additive Models (GAMs) were used to determine concordance with the BRT approach. Selection of the explanatory variables to include in GAMs was based on BRT results; only day of year and SST were investigated, both as smoothed continuous variables. As with BRTs, separate GAMs were carried out for each sex, with either binary daily female presence/absence or male presence/absence as the response variable. GAMs were built with a binomial error structure with log link function and maximum likelihood estimation, using the R *mgvc* package (Wood 2011).

#### 2.4.3 Interannual Temperature Trends and Temperature‐Linked Arrival Cues

The role of temperature in driving *S. squatina* presence in the study area—or more specifically in cueing movement into the area (study aim (iii))–was investigated based on the results from BRT and GAM models and on the interannual variability in female presence. SST was plotted alongside daily presence over four full years (2019–2023) to examine longer term climatic trends. Based on the observed interannual patterns of *S. squatina* presence, we examined and compared a “regular” breeding season (2021) (e.g., arrival of both sexes in mid‐to‐late autumn) and the “anomalous” breeding season (2022) (e.g., arrival of males but not females) in more detail, by plotting SST (and key SST values identified in environmental models) and SSTA against female presence for these periods (study aim (iv)).

## 3 Results

### 3.1 Acoustic Detection Summary

**FIGURE 2:**
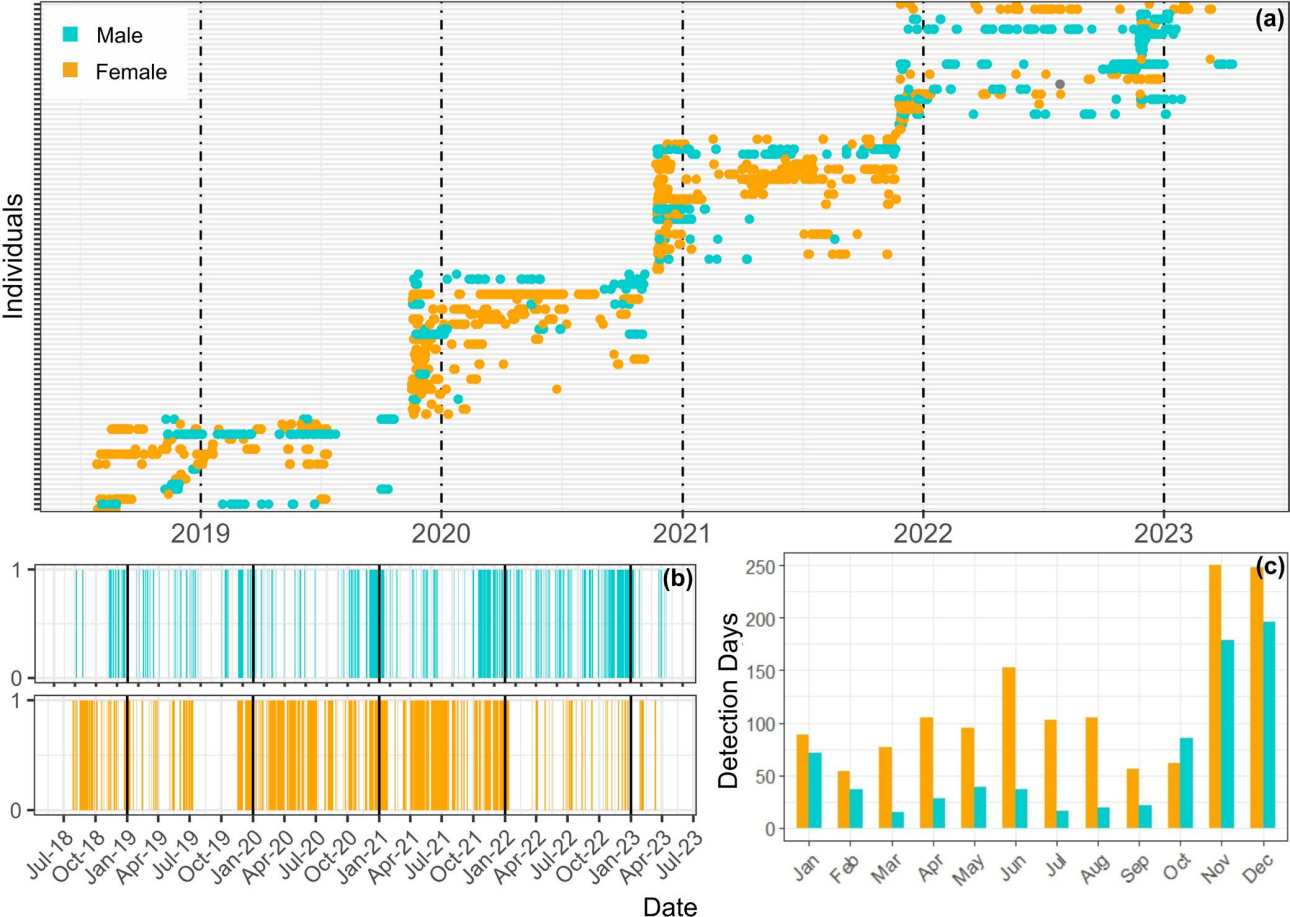
Time series plots of male and female *Squatina squatina* presence throughout the study period (July 2018–April 2023), shown as (a) detections over time for each individual (top) and (b) daily presence/absence (where orange or blue vertical lines indicate presence), and (c) detection days per months of the year.

A total of 158,397 acoustic detections were recorded between 27‐July 2018 and 14‐April 2023. Of the 112 tagged individuals, 90.2% (*n* = 101) were detected at least once. This included 92.1% of tagged males (*n* = 35) and 89.2% of tagged females (*n* = 66). The total number of days detected per individual varied from 1 to 77 in males and from 1 to 145 in females. As expected, there was strong seasonal variability in *Squatina squatina* presence for both sexes (Figure 2). Across the study period, on average, both male and female presence peaked in November and December, but females had a secondary peak in June and remained more consistently present throughout the rest of the year than males. This pattern of peaking presence was generally consistent across all study years, with the exception of 2022; in this year, the occurrence of female *S. squatina* was low throughout the year and did not peak. This was also reflected in the November 2022 tagging campaign, which showed an abnormal sex ratio with eight males and two females tagged (see Table 1).

### 3.2 Environmental Model Results

Optimal BRT model parameters for male and female *S. squatina* are included in Table 2. In both models, the full set of original explanatory variables was retained (SST, SSTA, chlorophyll‐a, dissolved oxygen, salinity, wind speed and day of year). The addition of Calima variables improved model performance for females, but not for males; specifically, the addition of Calima variables in female models increased explained deviance by 1.7% when compared to the best performing non‐Calima model. The final male model explained 31.2% of deviance and the final female model 39.5% of deviance. Training and CV AUC values indicated strong performance for both the male model (Training AUC = 0.983, CV AUC = 0.852) and female model (Training AUC = 0.999, CV AUC = 0.889) (Lane et al. 2009) (Table 2, Table S3).

**TABLE 2:** Summary of optimal parameters and evaluation metrics in the best performing male and female Boosted Regression Tree models.

		Male	Female
Input parameters	Number of trees	2300	2400
	Bagging fraction	0.7	0.6
	Tree complexity	5	8
	Learning rate	0.005	0.005
Evaluation metrics	Mean deviance	0.909	0.817
	Deviance explained (%)	31.2	39.5
	Training AUC	0.983	0.999
	Cross‐validation (CV) AUC	0.852	0.889
	Training AUC–CV AUC	0.131	0.110

**FIGURE 3:**
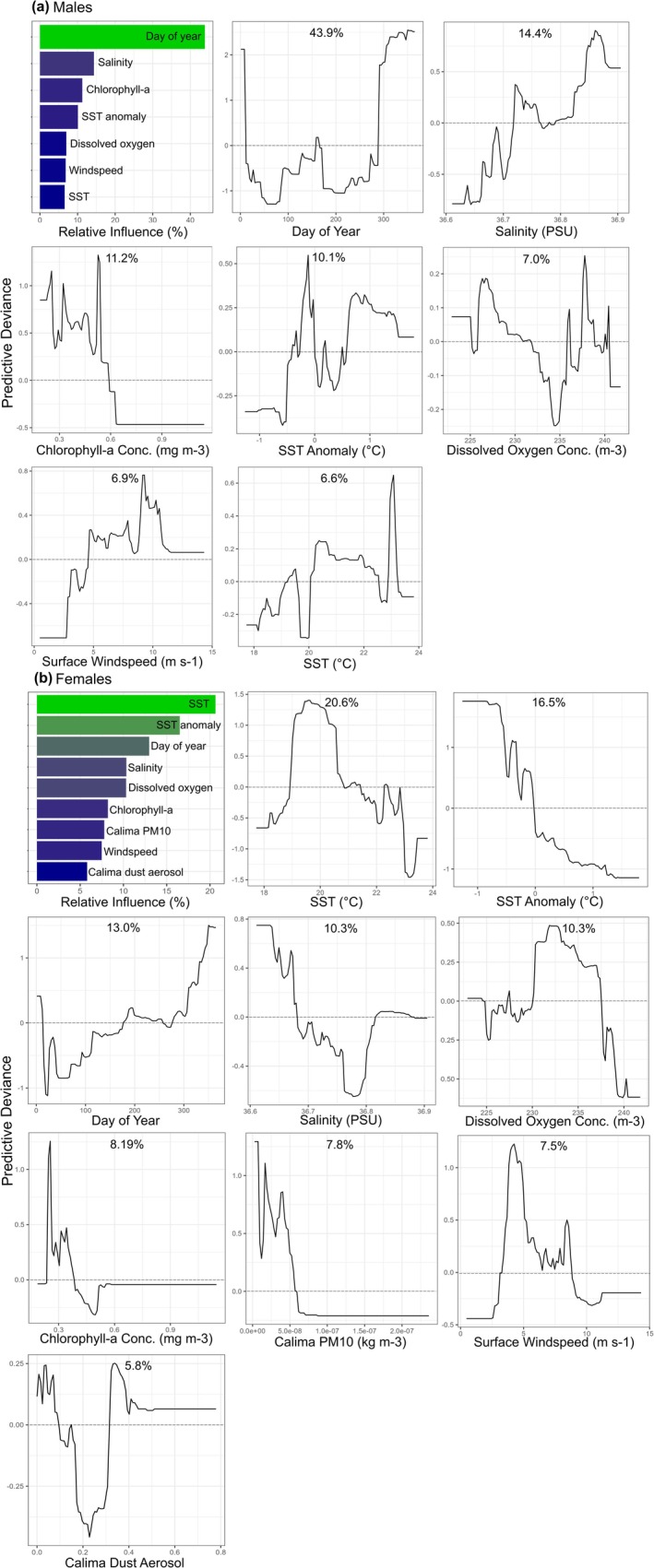
Boosted Regression Tree (BRT) model results for (a) males and (b) females, showing the relative influence of model predictor variables on presence, and partial effects plots of the relationships between predictor variables and *Squatina squatina* presence.

The relative influence of predictors varied greatly between sexes. For male *S. squatina* , day of year (doy) had by far the largest influence, contributing 43.9% (Figure 3a). Specifically, the probability of male occurrence rapidly increased at around doy 300 (late October), peaked at doy 331–335 (end of November) and remained high until around doy 10 (early January). With the exception of a small peak in predicted presence for just a few days in early June, the probability of male presence remained below 0 for the rest of the year. All other predictors had a relative influence of < 15%; salinity was the most influential environmental predictor (14.4%), followed by chlorophyll‐a concentration (11.2%), SSTA (10.1%), dissolved oxygen concentration (7.0%), wind speed (6.9%) and SST (6.6% each) (Figure 3a). For female *S. squatina* , SST and SSTA were the most influential predictors, contributing 20.6% and 16.5%, respectively (Figure 3b). Female occurrence peaked between approximately 19.1°C and 20.7°C (maximum = 19.6°C); above this temperature, occurrence rapidly decreased as SST increased. Above approximately 22.5°C, the probability of female presence remained below 0 (i.e., the model predicts female absence above this temperature). Overall, female presence decreased as SSTA increased, and the probability of presence dropped below 0 when the SSTA became positive. All other predictors had a relative influence of < 15%, with doy (13%) followed by salinity (10.3%), dissolved oxygen concentration (10.3%), chlorophyll‐a concentration (8.2%), PM10 (7.8%), wind speed (7.5%), and dust aerosol (5.8%) (Figure 3b).

**FIGURE 4:**
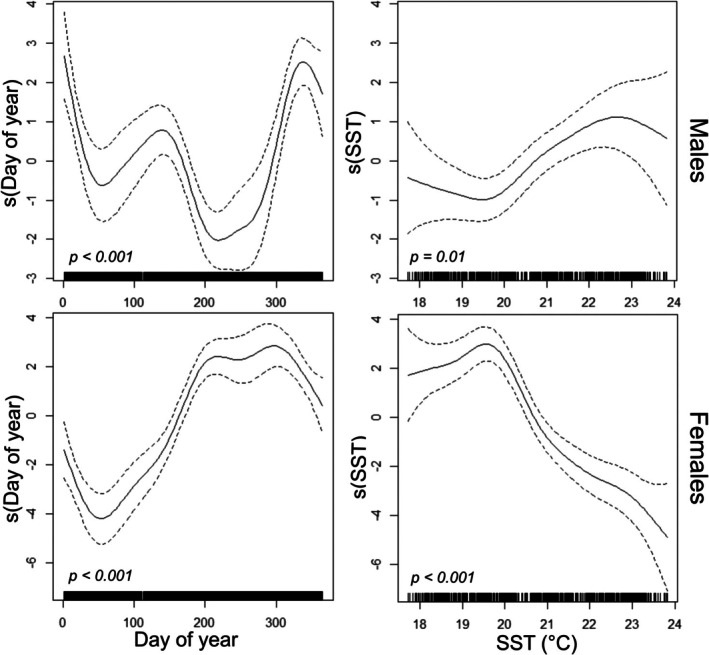
Generalised Additive Model (GAM) partial effects plots of the relationships between predictors day of year (doy) and sea surface temperature (SST), and presence of male *Squatina squatina* (top) and female *S. squatina* (bottom).

Based on BRT model results, day of year and SST were identified as variables of interest to be included in GAMs. The BRT results for SST were well corroborated by GAMs (Figure 4). SST was a weakly significant predictor of male presence (*p* = 0.01), which generally increased as SST increased above 20°C, and peaked at ~22.8°C. For females, SST was a strongly significant predictor of presence (*p* < 0.001), peaking at ~19.6°C and then decreasing as SST increased. Male and female GAMs explained 24.4% and 26.6% of deviance, respectively.

### 3.3 Temperature Trends and Cues

**FIGURE 5:**
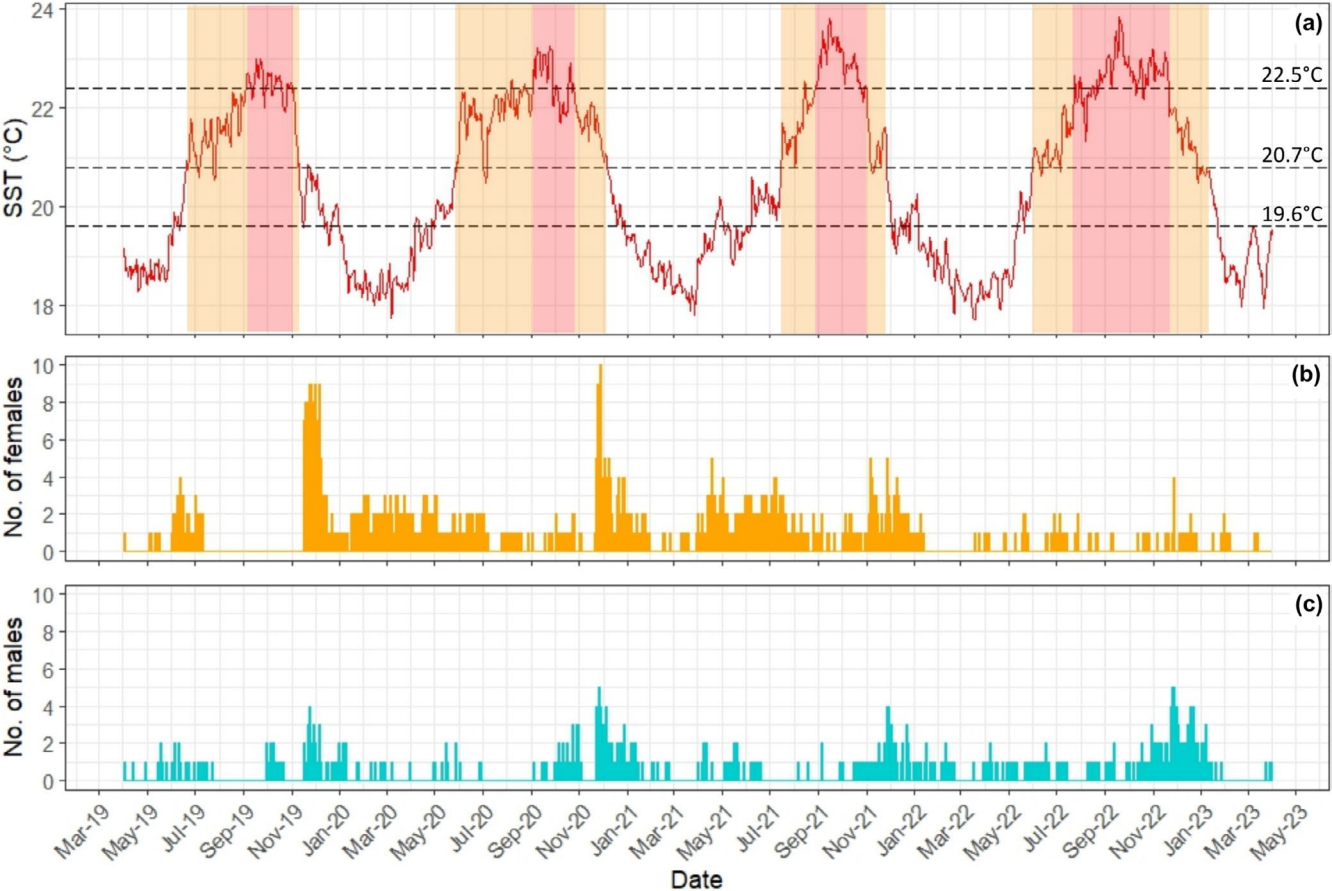
Time series of (a) daily average sea surface temperature (SST) and daily counts of (b) female and (c) male *Squatina squatina* in the study are between April 2019 and April 2023. Horizontal lines indicating key values identified in female environmental models; 19.6°C as peak presence, 20.7°C above which presence rapidly dropped, and 22.5°C above which the probability of presence remained below 0. In turn, orange shading indicates SST ranges between 20.7°C and 22.5°C, and red shading SSTs above 22.5°C.

Over the duration of the study, a general upward trend in peak SST and duration of high temperatures was observed (see S4 for full environmental time series). The timing of peak SST remained the same each year (mid‐September) but increased from 22.99°C in 2019 to 23.81°C in 2022. The total number of days with SST above 22.5°C increased from 30 days in 2019 to 85 days in 2022. In most years, both male and female *S. squatina* presence peaked in mid‐to‐late November, coinciding with the seasonal fall in SST (Figure 5b,c).

**FIGURE 6:**
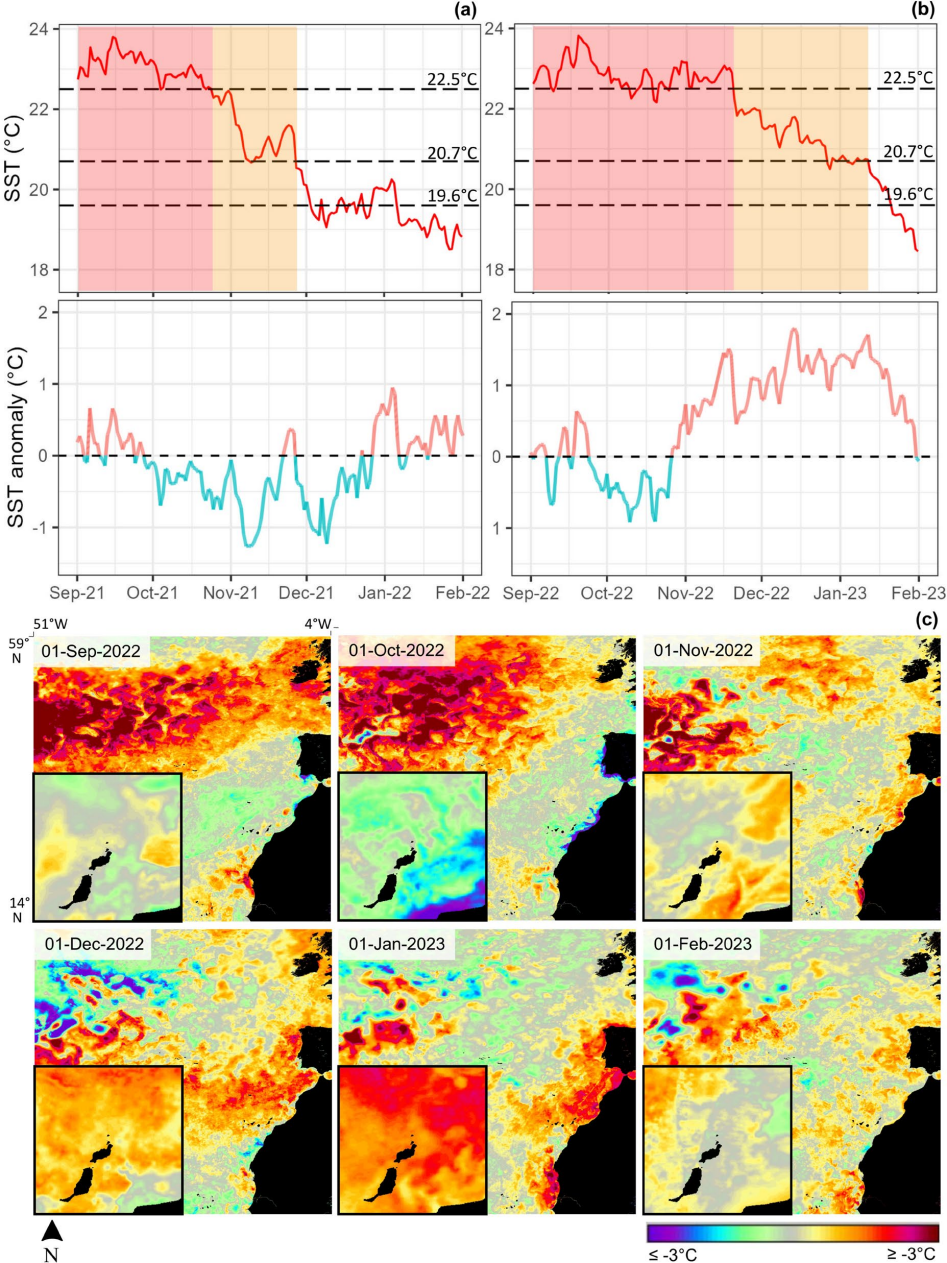
Daily mean sea surface temperature (SST) and SST anomaly (SSTA) over the (a) 2021 and (b) 2022 *Squatina squatina* breeding season with key SST and SST ranges labelled as in Figure 5, and (c) Maps showing the development of SSTA from September 2022 to February 2023, across the Northeast Atlantic region (larger maps) and northeast Canary Islands (inserts). Imagery for (c) is from the NASA Worldview application, accessible at https://worldview.earthdata.nasa.gov

In 2022, male presence peaked as usual, but female numbers remained low year‐round and did not markedly increase in autumn as in other years. Figure 6 compares SST and SSTA over the autumn‐winter breeding season in a “regular” year of *S. squatina* presence (2021) and the “anomalous” year (2022) where presence was unexpectedly low. Whereas in 2021, SST fluctuated between negative and positive anomalies, in 2022 a positive SSTA of up to ~1.8°C was present for the entirety of November and December, and January 2023 (Figure 6). Furthermore, while the annual peak SST value did not differ greatly between these years (23.80°C in 2021 and 23.81°C in 2022), the duration of high temperatures was extended in 2022, with SSTs above 22.5°C persisting into late November and falling approximately a month later than in the previous year.

## 4 Discussion

The present study utilized acoustic telemetry, satellite remote sensing, and environmental modelling to investigate environmental associations and drivers of seasonal presence in adult angelsharks, *Squatina squatina* , in La Graciosa Marine Reserve, Canary Islands. We found that females were more influenced by environmental parameters and had more distinctive climate‐driven cues in their behaviour than males. Temperature (SST and SSTA) had a particularly strong influence on females, with presence decreasing with increased SST and with increasingly positive SSTA. Throughout the study, an overall warming trend was recorded, with annual increases in maximum SST, duration of high SSTs, and occurrence of positive SST anomalies. We observed an anomaly in *S. squatina* presence in 2022, with marked absence of females throughout the year and particularly during the usual mating period. This coincided with unusual temperature patterns both locally and across the Northeast Atlantic region, with high SSTs persisting well into the breeding season and a strong positive SSTA remaining throughout the winter of 2022–2023.

### 4.1 Environmental Drivers: Sex Differences and Seasonal Cues

Modelling adult *S. squatina* presence in relation to a selection of environmental parameters showed that females were more strongly influenced by environmental conditions than males. Females were particularly influenced by temperature variables, with a general pattern of decreasing presence with increasing SST and SSTA. Models identified three key temperature values in relation to female presence; ~19.6°C at which presence peaked, ~20.7°C above which presence rapidly decreased, and ~22.5°C above which females were predicted to be absent. Although thermal thresholds have not been defined for this species, previous studies report similar relationships between temperature and *S. squatina* presence. Noviello et al. (2021) found that SST had greater influence on females than males, and Mead et al. (2023) found sex‐based habitat preference, with males more likely to utilize deeper offshore areas and females preferring shallow sheltered areas, possibly related to female use of warmer water for certain physiological processes (e.g., during parturition). Meyers et al. (2017) recorded presence of *S. squatina* in temperatures between 18°C and 22°C, roughly aligning with the values reported in this study.

In ectothermic elasmobranchs, sex‐based differences in environmental association can broadly be explained in terms of physiology, reproduction, and social behavioral dynamics (Wearmouth and Sims 2008). Physiologically, the sexes have different requirements, with females generally having more energetically demanding lifestyles in relation to reproductive processes such as gestation and live birth (Sims 2003). As such, females tend to have stronger thermal preferences (e.g., Wallman and Bennett 2006) in order to regulate metabolic processes and energy expenditure. For example, the use of warmer waters by mature females is commonly observed in elasmobranchs as a means of increasing body temperature and, in turn, increasing the rate of functions such as digestion, growth, and embryo development (Hight and Lowe 2007; Speed et al. 2012; Schlaff et al. 2014). Females may also use cooler water when energy needs to be conserved, such as during migration (e.g., McMillan et al. 2019). According to sexual conflict theory, males and females also have divergent reproductive strategies, which are reflected in behavior and habitat selection; crucially, traits and adaptations that increase fitness in one sex may be costly for the other sex (Chapman et al. 2003; Hosken and Stockley 2005). In response to the differing reproductive investment associated with anisogamy, males generally increase reproductive success by mating frequently, whereas females prioritize physical fitness for gestation and offspring survival; in turn, female fitness can be directly compromised by aggressive, energetically demanding male mating behavior (Sims et al. 2001; Wearmouth et al. 2012). As such, habitat selection in males is driven by maximizing female encounters and mating opportunities, and in females by avoidance of males and seeking environmental conditions optimal for reproductive physiology, as described above (Sims 2003; Darden and Croft 2008; Kimber et al. 2009). The finding that female *S. squatina* behavior is more affected by their environment than males may therefore be partly explained by divergent reproductive motivations, with females selecting habitat with preferred environmental conditions and males tracking expected seasonal female presence regardless of thermal and environmental changes.

Interestingly, Calima variables also had an influence—albeit small—on female presence. Although the geography of Calima and its impact on human health and infrastructure has been well reported (e.g., Ginnadaki et al. 2014; Al‐Hemoud et al. 2017), as has its impact on ocean biochemistry and geochemistry (e.g., Gallisai et al. 2014; Francis et al. 2022), very little information is available on its indirect effects on marine animals. Desert dust carries nutrients such as phosphorus, calcium, and iron, which often stimulate enhanced primary productivity when deposited in the ocean (Goudie and Middleton 2001). Rodríguez et al. (2023) showed that skipjack tuna, *Katsuwonus pelamis*, distribution correlates with that of Saharan dust plumes in the Northeast Atlantic, likely tracking the associated nutrients. Given the strong relationship between Calima and factors that are important for marine taxa including chondrichthyans—namely temperature and productivity—future research focused on Sahara dust and species distribution would be beneficial, especially as dust events are expected to intensify (Goudie and Middleton 2001).

The findings of this study support the hypothesis that adult *S. squatina* distribution and habitat use around the Canary Islands is seasonal (Meyers et al. 2017; Mead et al. 2023), and suggest that movement and redistribution of *S. squatina* is caused by seasonal environmental changes. Environmentally cued movement in chondrichthyans has been linked to wind speed and barometric pressure (e.g., Udyawer et al. 2013), upwelling events (e.g., Spurgeon et al. 2022), salinity (e.g., Morgan et al. 2017), and most commonly to temperature, often in the form of specific thermal thresholds or “switch‐points.” For example, Dudgeon et al. (2013) recorded seasonally cued arrival of zebra sharks, *Stegostoma tigrinum*, when water temperature increased to 22°C, and Reyier et al. (2014) observed rapid latitudinal movement in lemon sharks, *Negaprion brevirostris*, when temperature dropped below 15°C. Seasonal arrival of *S. squatina* in coastal waters in October and November coincides with dropping water temperatures. We suggest that a seasonal thermal cue exists in this system, and that it disproportionately influences female sharks in La Graciosa Marine Reserve. That is, females choose not to move into the study area to mate until temperatures drop, specifically below 22.5°C. Evidence for this theory, as well as the theory of sexually divergent reproductive strategy and habitat selection, was reinforced by the abnormal absence of females recorded in 2022.

### 4.2 Disrupted Thermal Cues in 2022: A Window Into Future Climate Change Impacts?

Model results and examination of SST and SSTA between years strongly indicate that the recorded *S. squatina* behavioural anomaly in 2022 was related to temperature; or specifically, duration of high temperature and persistence of high SST and positive SSTA later in the year. For example, in 2019, 2020, and 2021, SSTs fluctuated close to or exceeded 22.5°C for approximately 2 months each year, and consistently dropped back below this value in October. In 2022, however, SST exceeded 22.5°C for nearly 4 months, and did not drop until late November. This coincided with a strong positive SSTA, which persisted from late October 2022 until nearly February 2023. These extremes were part of a wider regional pattern of thermal anomalies and MHWs occurring across the North Atlantic throughout much of 2022 and into 2023 (Figure 6c).

We suggest two interlinking temperature‐related hypotheses for these observations. First, if coastal temperatures within the study area were above a thermal threshold, females may have been forced to select alternative habitat, prioritizing optimal physiology over encountering males. In turn, given that males were shown to be less influenced by environmental conditions, male *S. squatina* may have been more tolerant of abnormally high temperatures, or more willing to endure temperatures outside of their preferred thermal range in order to search for mates, potentially prioritizing mating over thermal optima (Spurgeon et al. 2022). Further, greater general mobility and activity levels in males (Mead et al. 2023) might facilitate regular movement into deeper waters for thermal refuge with minimal increase to usual energy expenditure. Second, it could be that temperatures did not drop sufficiently or at the expected time to cue female *S. squatina* movement, disrupting seasonal redistribution. However, an inherent limitation of acoustic telemetry is that tagged animals are only monitored in specific locations, with no information on presence or behavior outside of the detection range of each receiver. As such, there must be caution in interpreting the absence of female *S. squatina* in our detection data as absence from the entire area.

### 4.3 Predicting Future Impacts of Climate Change on *S. squatina*

Globally, continued increase in sea surface temperatures throughout the 21st century is projected with virtual certainty, with possible increases of two to four times current temperatures under the lowest emission scenario (RCP2.6) and five to seven times under the highest emission scenario (RCP8.5) (Bindoff et al. 2019; IPCC 2021). In turn, the frequency, duration, intensity, and spatial extent of MHWs are predicted to increase in all regions (Frölicher et al. 2018; IPCC 2022). In the Northeast Atlantic, significant slowing of the Atlantic Meridional Overturning Circulation (AMOC), ocean acidification, reduction in surface oxygen concentration, and reduced intensity of coastal upwelling are predicted (Olson et al. 2018; Kjesbu et al. 2022; Mishonov et al. 2024); the latter may result in enhanced warming and a globally significant loss of primary productivity in the Canary Current and upwelling system, as the transport of cold, nutrient‐rich waters to the surface is reduced (Pardo et al. 2011; Siemer et al. 2021). Ecosystem impacts are expected to be amplified for small islands and coastal zones, where sea level rise, storm surges, and extreme rainfall events will combine with local human disturbance, driving coral bleaching, loss of seagrass, mangrove die‐off, and beach erosion (Bindoff et al. 2019; IPCC 2022; Marrero‐Betancort et al. 2022).

From our findings, there are two key implications to consider around *S. squatina* response and adaptation to climate change. First, the differential risk and adaptation potential between sexes. Although there is relatively little research into sex differences in shorter‐term behavioral response and adaptation to environmental change (Brand et al. 2023; Gissi et al. 2023), studies have already identified divergent impacts on males and females in marine predators (e.g., Barbraud and Weimerskirch 2001; Ouled‐Cheikh et al. 2024). Sex differences in thermal tolerance and thermal safety margins are often explained by physiological requirements. For example, Missionário et al. (2022) hypothesized that lower thermal tolerance in female ditch shrimps, *Palaemon varians*, was due to greater energy expenditure in females relating to reproductive investment and larger body size. In the present study, we showed that female *S. squatina* were more strongly associated with temperature generally, and more responsive to thermal extremes in 2022. The greater site fidelity and lower activity levels in female *S. squatina* (Mead et al. 2023) may mean that movement to seek out preferred thermal environments is more energetically and ecologically costly for females than for males, but that thermal extremes may force this behavior in females. If the net result is divergent habitat use, reproductive mismatch, and increased sexual segregation during environmental extremes, this could be detrimental to breeding success. The most extreme possibility is that certain areas—including some areas within LGMR—become inhospitable to female *S. squatina* if temperature anomalies persist. Ultimately, failing to account for sex differences in response to environmental change can lead to reduced success of conservation efforts (Ellis et al. 2017; Gianuca et al. 2019; Gissi et al. 2023).

Second, environmental cues are expected to be increasingly disrupted under climate change (McNamara et al. 2011; Winkler et al. 2014). Under ‘normal’ conditions, cues are time‐sensitive and coincide with the optimal timing for particular life history activities, such as migration or breeding. If a shift in the nature or timing of formerly reliable cues—such as a seasonal drop in temperature—is sufficient to disrupt the relationship with optimal timing for activities such as mating, the cue essentially becomes outdated (McNamara et al. 2011; Anderson et al. 2013; Winkler et al. 2014). This can lead to mistiming of activities and impact breeding success and survival (McNamara et al. 2011). This has been observed across a range of taxa, including terrestrial herbivores (e.g., Post and Forchhammer 2008) and birds (e.g., Mayor et al. 2017), marine predators (e.g., Cherry et al. 2013) and fish (e.g., Dufour et al. 2010). If a seasonal temperature reduction provides a cue for inshore movement and coastal redistribution in *S. squatina* , it follows that this cue may be increasingly disrupted with projected environmental changes. As such, the observations of 2022–2023 may offer a window into future impacts of climate change on *S. squatina* in the northeast of the Canary Islands, and possibly further afield.

### 4.4 Implications for Conservation and Future Research

Within the last century, severe population decline and range contraction in *S. squatina* has primarily been driven by overexploitation, habitat degradation, and other anthropogenic impacts (Barker et al. 2022; Miller 2016; Gordon et al. 2017; Lawson et al. 2020). In the Canary Islands, many of these threats are ongoing, and conservation approaches to date have focused on addressing these concerns. The impacts and risks associated with climate change are still emerging but are likely to exacerbate and interact with existing stressors (Hare et al. 2016; IPCC 2022). For example, in a population known to sexually segregate (Mead et al. 2023), temperature‐driven shifts in space‐use may increase the possibility of differential exposure to fishing pressure between sexes. In turn, species vulnerability to climate change is increased by the presence of other stressors, especially at equatorward range boundaries where continued overfishing is hypothesised to speed up displacement (Chin et al. 2010; Lenoir et al. 2020). In future *S. squatina* conservation measures, especially for the Canary Islands populations at the upper thermal limit, it is critical that climate change adaptation is considered and actively incorporated.

Future approaches will need to consider adaptive management, whereby conservation measures account for environmental change and dynamic species responses (Hobday 2011; Braun et al. 2023). An approach for *S. squatina* could be static environment‐centered conservation, which can include traditional spatially fixed protected areas, but designed based on important physical or environmental features, rather than being species‐focused (Hobday 2011; Groves et al. 2012). In this case, priority should be given to identifying environments and features which might be robust to climate change, and/or which are already recognized as thermal refugia (Keppel et al. 2012; Groves et al. 2012). Given the growing body of evidence suggesting that adult *S. squatina* utilize deeper offshore waters than previously thought (e.g., Mead et al. 2023), as well as the general climate‐driven vertical habitat shift observed in other elasmobranchs (e.g., Coulon et al. 2024), it may be important to consider protection of cool deep habitat around the Canary Islands. A second approach is dynamic management and temporary measures, which are only activated during short‐term extremes such as MHWs (Cheung and Frölicher 2020), aimed at alleviating other pressures (e.g., fishing, recreational water‐use) during—or in the months following—periods of elevated thermal stress. This could operate in conjunction with—or be an extension of—temporal conservation measures such as restriction of certain fisheries during a defined period in a critical area. However, dynamic management requires continuous, long‐term planning and monitoring, as well as substantial collaborative effort between multiple actors (e.g., scientists, fishers, government bodies) which is logistically challenging (Hobday 2011; Reside et al. 2018).

Crucially, these conservation approaches require further data. In this study, we explored the direct influence of environmental conditions on ectotherm physiology and behaviour. Indirect biological factors such as prey availability and distribution are also inherently linked to habitat selection and distribution in marine predators (Sims et al. 2006; Florko et al. 2023). Indeed, we suggest that the absence of prey‐related variables may account for some of the missing predictive power in our models of *S. squatina* presence. Given that prey species distribution shifts, decoupling of predator–prey phenology and trophic reorganisation are understood to be key impacts of oceanographic change (Burthe et al. 2012; Durant et al. 2019; Gallagher et al. 2022), it will be important to investigate how diet and foraging ecology interact with environmental cues in higher trophic level species such as *S. squatina* . Further, a particularly key knowledge gap remains in our understanding of seasonal adult *S. squatina* habitat use and movement: that is, where exactly do *S. squatina* go outside of the coastal breeding season? There is a strong evidence‐based hypothesis that most *S. squatina* utilise coastal habitat for mating, parturition, and pupping, and redistribute offshore outside of this period. However, in this study—as in all studies—direct observations were made only of the coastal, inshore portion of the species' life history. In the context of understanding environmental cues and thermal refugia, this leaves a significant data gap. Seasonally cued migration—as is hypothesised for *S. squatina* —relies not only on coupling between cue and activity but also between different locations along a migratory pathway; that is, coupling between the cue that initiates migration in the start‐location and the habitat conditions at the destination (Anderson et al. 2013; Winkler et al. 2014; Chmura et al. 2019). Due to spatiotemporal variation in environmental change, it is possible for conditions at different parts of an organism's distribution or migratory pathway to become uncoupled; for example, if temperature increases more rapidly in one location than in the other (Anderson et al. 2013; Winkler et al. 2014). We therefore suggest that an urgent research priority is broader‐scale monitoring of *S. squatina* , with the aim of obtaining year‐round, spatially complete data on habitat use and environmental tolerance. Only then can the full extent of climate change impacts on this Critically Endangered species be understood, monitored, and adapted to.

## 5 Conclusion

With unprecedented warming and oceanographic change continuing across the Northeast Atlantic Ocean, understanding and predicting marine species and ecosystem response to climate change in this region—and indeed globally—are urgent priorities. This study demonstrates how environmental change and thermal extremes have already altered the behavior and distribution of an ectothermic marine predator, the angelshark, *Squatina squatina*, with greater environmental and thermal sensitivity in females leading to sexually divergent habitat use and impacting breeding behavior. Our findings show that this Critically Endangered species of global conservation interest is more acutely vulnerable to climate change than previously thought, starkly highlighting the importance of species‐specific, real‐time environmental and behavioral data. These findings also highlight the need to prioritize and incorporate such data into new and existing conservation measures, so that efforts to protect threatened marine ectotherms remain ecologically relevant in a rapidly warming ocean.

## Author Contributions

**Lucy R. Mead:** conceptualization, data curation, formal analysis, funding acquisition, investigation, methodology, project administration, resources, visualization, writing – original draft, writing – review and editing. **Adam Piper:** conceptualization, methodology, supervision, writing – review and editing. **David Jiménez Alvarado:** conceptualization, data curation, funding acquisition, investigation, methodology, project administration, resources, supervision, writing – review and editing. **Eva Meyers:** conceptualization, data curation, funding acquisition, investigation, methodology, project administration, supervision, writing – review and editing. **Joanna Barker:** conceptualization, data curation, funding acquisition, investigation, methodology, project administration, supervision, writing – review and editing. **Hector Toledo‐Padilla:** conceptualization, data curation, funding acquisition, investigation, project administration, writing – review and editing. **Edy Herraiz:** conceptualization, investigation, resources. **Alberto F. Campoamor:** conceptualization, investigation, resources. **Michael Sealey:** conceptualization, investigation. **Maria Belén Caro:** conceptualization, investigation. **Tomàs Bañeras:** investigation, project administration. **Charlotte Pike:** investigation. **Matthew Gollock:** conceptualization, investigation, supervision, writing – review and editing. **Felipe Ravina‐Olivares:** investigation. **David M. P. Jacoby:** conceptualization, data curation, funding acquisition, investigation, methodology, project administration, resources, supervision, writing – review and editing.

## Conflicts of Interest

The authors declare no conflicts of interest.

## Supporting information


Data S1.

## Data Availability

The data and code that support the findings of this study are openly available via Zenodo at https://doi.org/10.5281/zenodo.15682052. NASA GHRSST sea surface temperature data are available from the Jet Propulsion Laboratory (JPL) Physical Oceanography Distributed Active Archive Center (PO.DAAC) at https://doi.org/10.5067/GHGMR‐4FJ04. NOAA ocean colour and sea surface wind speed data are available from NOAA Coastwatch at https://coastwatch.noaa.gov/erddap/griddap/noaacwNPPN20S3ASCIDINEOF2kmDaily.html (no data DOI available) and https://doi.org/10.3389/fmars.2022.935549, respectively. Copernicus salinity data and dissolved oxygen concentration data are available from the Copernicus Marine Service (CMS) at https://doi.org/10.48670/moi‐00027 and https://doi.org/10.48670/moi‐00026, respectively. Copernicus Calima dust data are available from the Copernicus Atmospheric Monitoring Service (CAMS) Atmospheric Data Store at https://doi.org/10.24381/04a0b097.
