# Supplementary material for: Rapid Ocean Warming Drives Sexually Divergent Habitat Use in a Threatened Predatory Marine Ectotherm
Source: Glob Chang Biol. 2025 Jul 16;31(7):e70331. doi: 10.1111/gcb.70331 (PMC12266942; doi:10.1111/gcb.70331)
Supplement: Supplementary file 1 — Data S1. [file GCB-31-e70331-s001.pdf]

Supporting Information

Variable	Description	Units	Spatial res.	Publisher	Data access	Reference
sst	Sea surface temperature	°C	0.01 x 0.01 DD	NASA JPL	ERDDAP in R	JPL MUR MEaSUREs Project 2015; Chin *et al.* 2017
sst_anom	Sea surface temperature anomaly	°C	0.01 x 0.01 DD	NASA JPL	ERDDAP in R	JPL MUR MEaSUREs Project 2015; Chin *et al.* 2017
chlora	Chlorophyll-a concentration	mg m^{-3}	2.32 x 2.32 km	NOAA CoastWatch	ERDDAP Griddap	Liu and Wang 2023
salinity	Sea water salinity	PSU	0.028 x 0.028 DD	CMS	CMS Data Store; QGIS	Soltillo *et al.* 2015
oxygen	Sea water dissolved molecular oxygen concentration	mmol m^{-3}	0.028 x 0.028 DD	CMS	CMS Data Store; QGIS	Gutknecht *et al.* 2019
windspeed_ms	Ocean surface wind speed	m s^{-1}	0.25 x 0.25 DD	NOAA NCEI	ERDDAP Griddap	Saha and Zhang 2022
calima_dust	Atmospheric dust aerosol optical depth at 550nm	na	0.01 x 0.01 DD	CAMS	CAMS Data Store; QGIS	
calima_pm	Atmostpheric particulate matter d < 10 µm	kg m^{-3}	0.4 x 0.4 DD	CAMS	CAMS Data Store; QGIS	

S1. Details of environmental variables included in models.
Acronyms: National Aeronautics and Space Administration (NASA); Jet Propulsion Laboratory (JPL); National Oceanic and Atmospheric Administration (NOAA); Copernicus Marine Service (CMS); Copernicus Atmosphere Monitoring Service (CAMS); National Centres for Environmental Information (NCEI).

	SST	SST_anom	Chlor-a	Oxygen	Salinity	Windspeed	Calima_dust	Calima_pm
SST	1							
SST_anom	-0.012	1						
Chlor-a	-0.46	0.04	1					
Oxygen	-0.9	0.08	0.37	1				
Salinity	-0.07	0.09	0.28	-0.13	1			
Windspeed	-0.22	-0.24	-0.08	0.51	-0.11	1		
Calima_dust	0.08	-0.01	-0.009	-0.05	-0.03	0.01	1	
Calima_pm	-0.24	-0.04	0.28	0.15	0.12	0.17	0.53	1

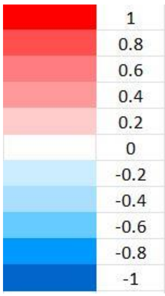

S2. Correlation matrix for Boosted Regression Tree input variables.

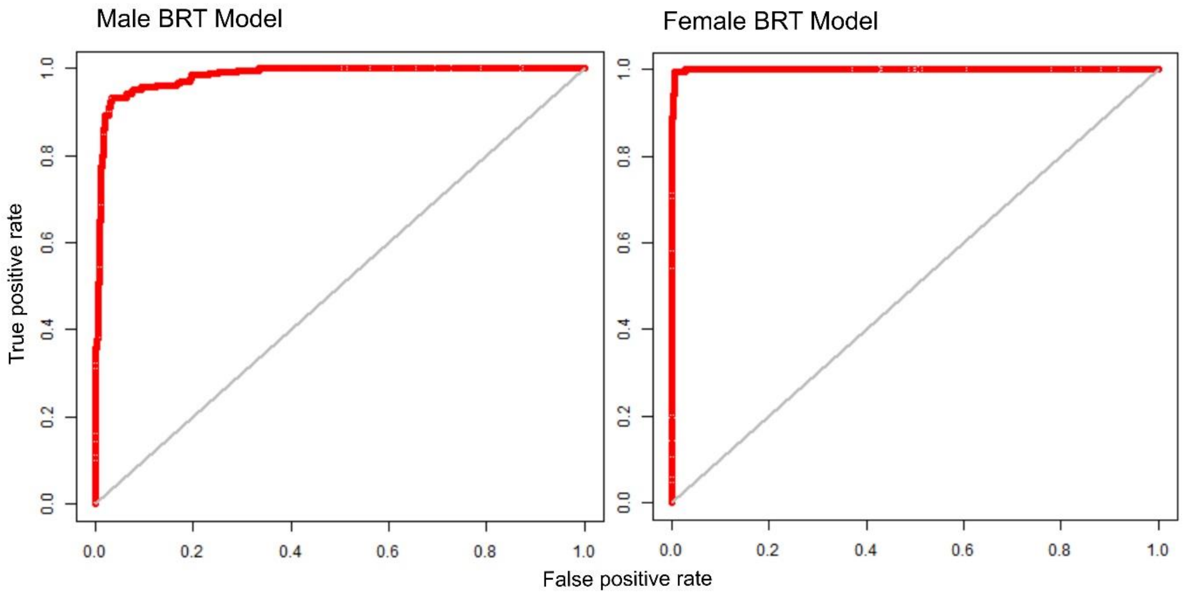

S3. Area Under Curve plots for the best performing male and female Boosted Regression Tree models.

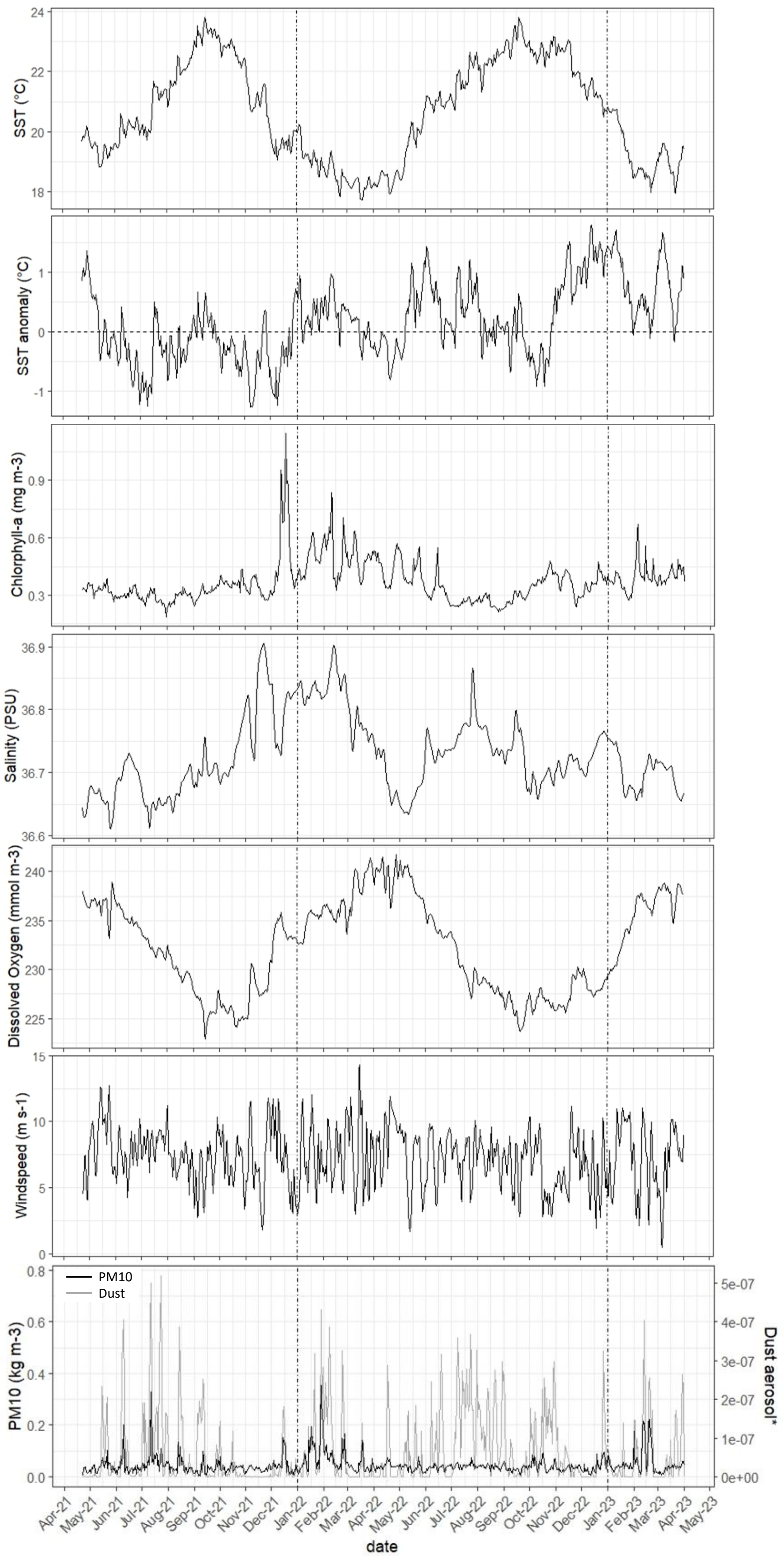

S4. Full time series plots for each environmental variable including in Boosted Regression Tree models.
